# Fronto-striato-thalamic circuit connectivity and neuromelanin in schizophrenia: an fMRI and neuromelanin-MRI study

**DOI:** 10.1038/s41537-023-00410-8

**Published:** 2023-11-10

**Authors:** Sunah Choi, Minah Kim, Taekwan Kim, Eun-Jung Choi, Jungha Lee, Sun-Young Moon, Sang Soo Cho, Jongho Lee, Jun Soo Kwon

**Affiliations:** 1 Department of Brain and Cognitive Sciences, Seoul National University College of Natural Sciences, Seoul, Republic of Korea; 2 Department of Neuropsychiatry, Seoul National University Hospital, Seoul, Republic of Korea; 3 Department of Psychiatry, Seoul National University College of Medicine, Seoul, Republic of Korea; 4 Department of Bio and Brain Engineering, Korea Advanced Institute of Science and Technology, Daejeon, Republic of Korea; 5 Department of Electrical and Computer Engineering, Seoul National University, Seoul, Republic of Korea; 6 Department of Psychiatry, Seoul National University Bundang Hospital, Seongnam, Republic of Korea; 7 Institute of Human Behavioral Medicine, SNU-MRC, Seoul, Republic of Korea

**Keywords:** Neuroscience, Schizophrenia

## Abstract

Changes in dopamine and fronto-striato-thalamic (FST) circuit functional connectivity are prominent in schizophrenia. Dopamine is thought to underlie connectivity changes, but experimental evidence for this hypothesis is lacking. Previous studies examined the association in some of the connections using positron emission tomography (PET) and functional MRI (fMRI); however, PET has disadvantages in scanning patients, such as invasiveness. Excessive dopamine induces neuromelanin (NM) accumulation, and NM-MRI is suggested as a noninvasive proxy measure of dopamine function. We aimed to investigate the association between NM and FST circuit connectivity at the network level in patients with schizophrenia. We analysed substantia nigra NM-MRI and resting-state fMRI data from 29 schizophrenia patients and 63 age- and sex-matched healthy controls (HCs). We identified the FST subnetwork with abnormal connectivity found in schizophrenia patients compared to that of HCs and investigated the relationship between constituting connectivity and NM-MRI signal. We found a higher NM signal (*t* = −2.12, *p* = 0.037) and a hypoconnected FST subnetwork (FWER-corrected *p* = 0.014) in schizophrenia patients than in HCs. In the hypoconnected subnetwork of schizophrenia patients, lower left supplementary motor area-left caudate connectivity was associated with a higher NM signal (β = −0.38, *p* = 0.042). We demonstrated the association between NM and FST circuit connectivity. Considering that the NM-MRI signal reflects dopamine function, our results suggest that dopamine underlies changes in FST circuit connectivity, which supports the dopamine hypothesis. In addition, this study reveals implications for the future use of NM-MRI in investigations of the dopamine system.

## Introduction

Schizophrenia is closely related to dopamine impairment. This relationship became known through the discovery of antipsychotic drugs targeting dopamine receptors, and all licenced antipsychotic drugs used in clinical practice today affect the dopamine system^1,2^. In postmortem studies, the concentrations of dopamine were increased in schizophrenia patients^3,4^. Recent positron emission tomography (PET) and single photon emission computed tomography (SPECT) studies reported robust increases in in vivo striatal dopamine synthesis and release^5,6^. According to the dopamine hypothesis, the most influential hypothesis regarding schizophrenia, schizophrenia occurs when increased striatal dopamine caused by various risk factors induces functional impairment^7^.

Dopamine changes in the midbrain are presumed to cause dysfunctions through connectivity changes in the fronto-striato-thalamic (FST) circuit^8,9^. In the FST circuit, which links the frontal cortex, basal ganglia, and thalamus, dopaminergic signalling from the midbrain affects the cortex via basal ganglia regions, including the striatum, substantia nigra (SN), and globus pallidus^10,11^. Functional connectivity alterations in the FST circuit have been reported in schizophrenia^9,12^. Changes in striatal connectivity are one of the most prominent findings in schizophrenia^13,14^. Previous studies speculated the involvement of dopamine in the underlying pathophysiology of dysconnectivity given the characteristics of the structures^12^. However, functional MRI (fMRI)-only studies cannot show a direct association with molecules.

Previous studies have attempted to reveal the association between dopamine function and FST circuit connectivity using both PET and fMRI^9,15–18^. In multimodal studies, there were associations between dopamine synthesis capacity and cortico-thalamic and fronto-striatal connectivity and between dopamine receptor density and striatal connectivity in schizophrenia patients^14,19,20^. However, prior investigations examined the association only for limited connections. In particular, basal ganglia regions with a small volume were excluded due to inaccurate delineation^21^. Therefore, further studies are needed to examine the association at the network level, including the small basal ganglia regions. In addition, PET imaging has several disadvantages, such as radioactive substance use, long acquisition time, and limited accessibility, that make it difficult to scan patients. Hence, alternative molecular imaging methods can contribute to further investigations of patients.

Neuromelanin-MRI (NM-MRI) is a noninvasive proxy measure of dopamine function^22,23^. Neuromelanin (NM) is synthesised by the oxidation of cytosolic dopamine and thus accumulates in the midbrain^24^. In nonneurodegenerative conditions, NM is produced as an alternative to excess dopamine and plays a neuroprotective role^25,26^. Preclinical studies have shown that increased dopamine results in NM accumulation in the SN^27^. NM-MRI captures paramagnetic NM-iron complexes, the form in which NM is present in cells, and the signal is proportional to the concentration of NM^22^. Furthermore, the NM-MRI signal in the SN is correlated with PET dopamine measures, including striatal dopamine release^22,28,29^. Taken together, the NM-MRI signals reflect dopamine activity in the nigro-striatal pathway^22^.

In this study, we aimed to investigate the association between FST circuit functional connectivity and NM in schizophrenia patients using fMRI and NM-MRI. We examined for the first time the association between dopamine system measures and individual connectivity in the FST circuit at the network level in schizophrenia. Here, we used NM-MRI considering its advantages over traditional molecular imaging methods, such as its noninvasiveness. Given that the NM-MRI signal reflects dopamine function, the association can contribute to the demonstration of the dopamine hypothesis. For this aim, we identified dysconnected subnetworks in the FST circuit and examined the relationship between its constituting connectivity and the NM-MRI signal. We hypothesised that schizophrenia patients would show changes in NM-MRI signal and FST circuit connectivity and that there would be a significant association between them.

## Methods

### Participants

We acquired data from 30 schizophrenia patients and 64 age- and sex-matched healthy controls. Schizophrenia patients were recruited from the inpatient and outpatient clinics of the Department of Neuropsychiatry and Seoul Youth Clinic (www.youthclinic.org), a centre for the prospective and longitudinal investigation of people at high risk for schizophrenia, at Seoul National University Hospital (SNUH). In this study, schizophrenia patients were subjects who were diagnosed with schizophrenia according to the Structured Clinical Interview for DSM-IV Axis I Disorders (SCID-I)^30^. Symptom severity was assessed using the Positive and Negative Symptom Scale (PANSS)^31^. The Global Assessment of Functioning (GAF) scale was used to evaluate the overall functioning of schizophrenia patients. The Hamilton Rating Scale for Depression (HAMD)^32^ and the Hamilton Rating Scale for Anxiety (HAMA)^33^ were used to evaluate the severity of participants’ depression and anxiety. Healthy controls were recruited via internet advertisements. To screen for the presence of psychiatric disorders or symptoms, healthy controls were evaluated using the Structured Clinical Interview for DSM-IV-Non-Patient Version (SCID-NP). Healthy controls with a past or current axis-I diagnosis and any first- to third-degree biological relatives who had a lifetime history of psychotic disorders were excluded from this study. The subjects’ intelligence quotient (IQ) was measured with the Korean version of the Wechsler Adult Intelligence Scale (K-WAIS)^34^.

The exclusion criteria for all participants included evidence of neurological disease or clinically significant head injury, substance abuse or dependence (except for nicotine), or intellectual disability (IQ < 70). Written informed consent was obtained from all participants and the parents of subjects younger than 18 after a full explanation of the procedures was provided (IRB No. H-1110-009-380, 1905/001-010). This study was conducted according to the Declaration of Helsinki (2013) and approved by the Institutional Review Board of SNUH (IRB No. H-2210-123-1371).

### Image acquisition and preprocessing

We acquired anatomical, resting-state functional, and NM images using a 3 T MRI scanner (Siemens Magnetom Trio). T1-weighted anatomical images were scanned using a magnetisation-prepared rapid gradient echo sequence (voxel dimension 0.8 mm isotropic, repetition time/echo time 2400/2.19 ms, flip angle 8°, and slices 224). Resting-state functional images were acquired for 6 min and 44 s using a gradient echo planar imaging pulse sequence (voxel dimension 2.3 mm isotropic, repetition time/echo time 1500/30 ms, multi-band acceleration factor 4, flip angle 85°, and slices 64). Field map images consisting of echo planar imaging data with opposite phase encoding directions (right-to-left and left-to-right) were also collected for susceptibility distortion correction for functional images (voxel dimension 2.3 mm isotropic, repetition time/echo time 4200/30 ms, flip angle 85°, and slices 64). During the fMRI session, participants were instructed to remain relaxed, keep their eyes closed, and not fall asleep. We scanned NM-sensitive images using a 3D gradient echo sequence (voxel dimension 0.8 × 0.8 × 1.5 mm^3^, repetition time/echo time 80/4.94 ms, flip angle 25°, and slices 32). Before each excitation, a magnetisation transfer pulse was applied to enhance the NM contrast^35^.

We preprocessed the brain imaging data using ENIGMA HALFpipe version 1.2.1 (https://github.com/HALFpipe)^36^, a semiautomated pipeline that relies on fMRIPrep^37^. The pipeline included susceptibility distortion estimation, spatial normalisation, grand mean scaling, independent component analysis (ICA)-based denoising, and temporal filtering. For spatial normalisation, the MNI152NLin2009cAsym template (2 mm) was defined as the standard space. Grand mean scaling was applied with a mean value of 10,000, and denoising was performed with the ICA-based automatic removal of motion artefacts (ICA-AROMA) method. Temporal filtering was conducted using a frequency-based temporal filter (0.01–0.1 Hz). Quality assessment was performed according to the ENIGMA HALFpipe quality control manual, and two subjects were excluded from further analyses due to high motion (mean framewise displacement (FD) > 0.5 mm, maximum FD > 3 mm).

### ROI definition and rs-fMRI analysis

**Fig. 1:**
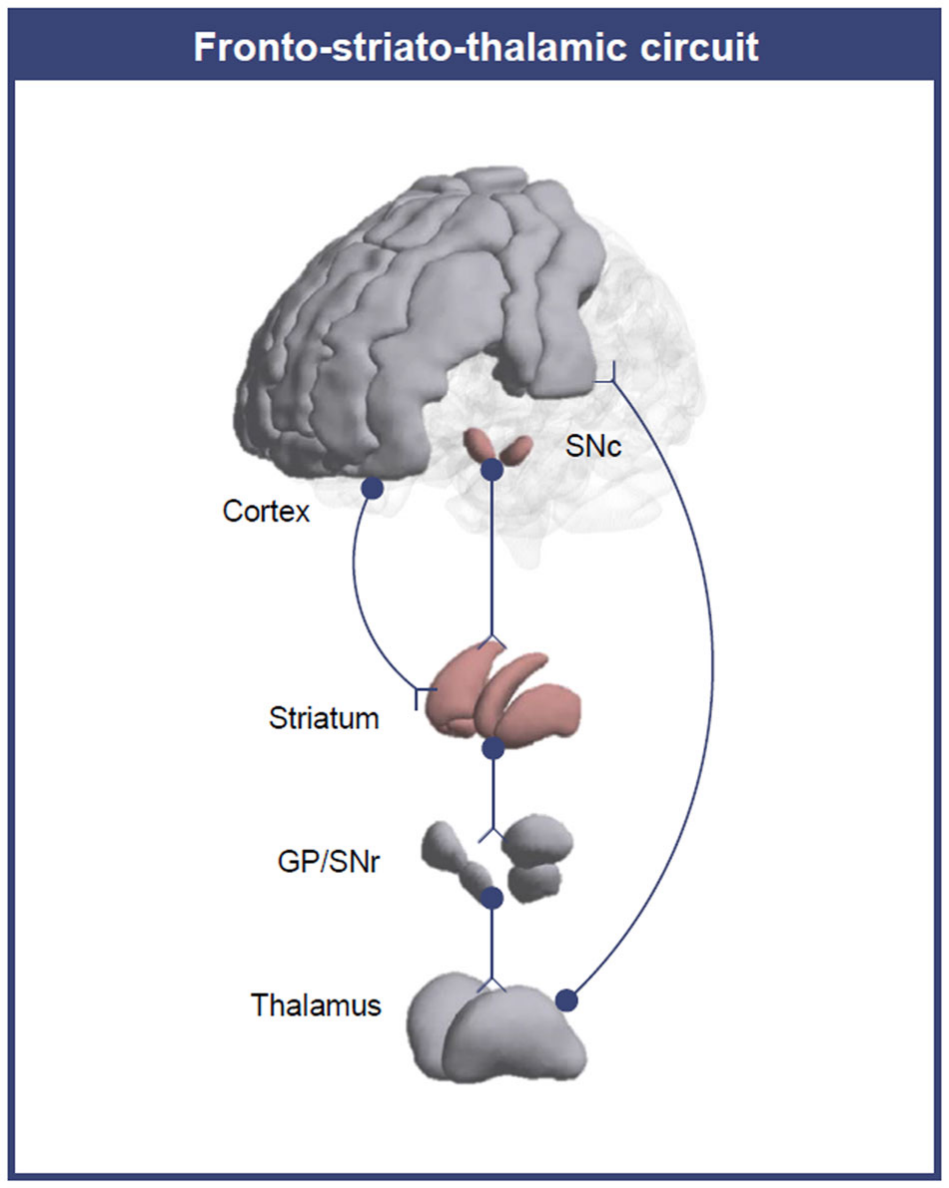
Fronto-striato-thalamic circuit. The dopamine signal from the substantia nigra pars compacta (SNc) projects to the striatum. The information flows to the globus pallidus (GP) and substantia nigra pars reticulata (SNr), then to the thalamus, and finally to the cortex. Excessive dopamine in the SNc interferes with subsequent signalling in the circuit.

We created an FST atlas spanning the bilateral frontal cortex, striatum, thalamus, globus pallidus, and SN using 3 different atlases (Fig. 1). For the frontal cortex, masks constituting the sensorimotor cortex (precentral gyrus, supplementary motor area (SMA), and postcentral gyrus), dorsolateral prefrontal cortex (dorsolateral superior frontal gyrus and middle frontal gyrus), and ventromedial prefrontal cortex (medial superior frontal gyrus and medial orbital superior frontal gyrus) were derived from the automatic anatomical labelling atlas 2^38^. The thalamus, caudate, putamen, and accumbens masks were obtained from the Harvard-Oxford subcortical atlas. For the globus pallidus, external and internal globus pallidus masks from the Pauli et al. (2018) atlas were combined. SN pars compacta and SN pars reticulata masks were also derived from the Pauli et al. (2018) atlas. The ROI masks were combined to create an FST atlas consisting of 28 regions.

Atlas-based connectivity analysis was performed in HALFpipe version 1.2.1, and time series were extracted from the 28 nodes of the FST atlas. Functional connectivity between pairs of nodes was estimated by calculating the Pearson correlation coefficient. As a result, a pairwise connectivity matrix between atlas regions was calculated for each participant.

### Functional network analysis

We tested whether there are dysconnected subnetworks in the FST circuit in schizophrenia patients using the network-based statistics (NBS) approach, a nonparametric statistical method to control the familywise error rate (FWER) when conducting mass univariate hypothesis testing^39,40^. Using the NBS toolbox version 1.2 (http://www.nitrc.org/projects/nbs/), we first conducted a mass univariate two-sample *t*-test on each connection with the primary threshold of *t* = 2.5 (corresponding to *p* = 0.01) to identify suprathreshold edges. To validate the robustness of our results, we additionally repeated the analysis across different primary thresholds (*t* = 2.1–2.9 corresponding to *p* = 0.04–0.005). Next, we yielded an empirical null distribution of the size of the largest network components by permuting data 5000 times. In each permutation, data were randomly relabelled into two groups, and suprathreshold connections were defined at the same statistical threshold. The FWER-corrected *p*-value was then calculated as the proportion of permutations for which the largest component size was the same or greater. Because we had focal interest confined to the FST circuit, we used the component intensity (i.e., the sum of test statistic values across all connections) rather than the component extent as the measure of component size.

### NM-MRI analysis

NM images were analysed using the manual segmentation method following Wang et al. (2018) in FSLeyes version 1.0.13^41^. First, three contiguous slices with the greatest SN area in the middle slice were selected. Then, 3 mm diameter circular masks were drawn at the lateral and medial parts of the SN. As a reference region, 4.5 mm diameter circular masks were drawn at the crus cerebri (CC). As a result, a total of 12 (4*3) and 6 (2*3) masks were drawn for the SN and CC, respectively. Next, we calculated the contrast ratio (CR) using the average signal intensity of SN (SI_SN_) and CC (SI_CC_) with the following equation: CR = (SI_SN_ – SI_CC_)/SI_CC_. Following prior NM-MRI studies^42,43^, we applied a thresholding method to define high-intensity NM-containing voxels in the SN. We used the upper quartile voxel values when calculating the SI_SN_. We used the CR value in subsequent analyses. The measurements were performed in a blinded manner.

### Statistical analysis

The statistical analyses were conducted using SPSS version 25. Demographic differences were tested using a *t*-test or chi-square test. We examined NM CR differences between groups using a *t*-test. The 95% confidence interval (CI) of the mean NM CR was calculated, as well as the mean in each group. Based on the NBS analysis results, we performed a multiple linear regression analysis to find specific subnetwork connections accounting for the NM level. In the analysis, the independent variables were abnormal subnetwork connections in schizophrenia, while the dependent variable was the NM level. We used the stepwise method for variable selection, which iteratively adds or removes independent variables based on their statistical significance.

## Results

### Demographics

We used data from 29 schizophrenia patients (average age 25.55 years, male 48.28%) and 63 healthy controls (24.13 years, 47.62%) in the analysis (Table 1). There were no significant differences in age, sex, or handedness between groups, although the IQ of healthy controls was higher than that of patients (*t* = 6.01, *p* < 0.001).

**Table 1:** Participants’ demographics and clinical characteristics.

	HCs (*n* = 63)	SCZ (*n* = 29)	*t* or *X*^2^	*p*
Age (years)^a^	24.13 (3.70)	25.55 (4.43)	−1.61	0.110
Sex (male/female)	30/33	14/15	0.00	0.953
Handedness (right/left)	56/7	24/4	0.18	0.668
IQ^a^	127.55 (7.77)	102.64 (17.58)	6.91	<0.001*
DOI (months)^a^		73.52 (46.70)		
PANSS^a^				
Positive symptoms		11.48 (3.09)		
Negative symptoms		13.33 (4.59)		
General symptoms		24.04 (4.70)		
GAF^a^		56.37 (13.87)		
HAMA^a^		2.96 (2.41)		
HAMD^a^		3.96 (2.95)		
Antipsychotic dose^b^		13.65 (8.96)		

*HCs* healthy controls, *SCZ* schizophrenia patients, *IQ* intelligence quotient, *DOI* duration of illness, *PANSS* Positive and Negative Syndrome Scale, *GAF* Global Assessment of Functioning, *HAMA* Hamilton Rating Scale for Anxiety, *HAMD* Hamilton Rating Scale for Depression.

^a^The values are presented as the mean (standard deviation).

^b^Mean daily olanzapine equivalent of antipsychotics. (*) indicates significance at *p* **<** 0.05.

### NM alterations

**Fig. 2:**
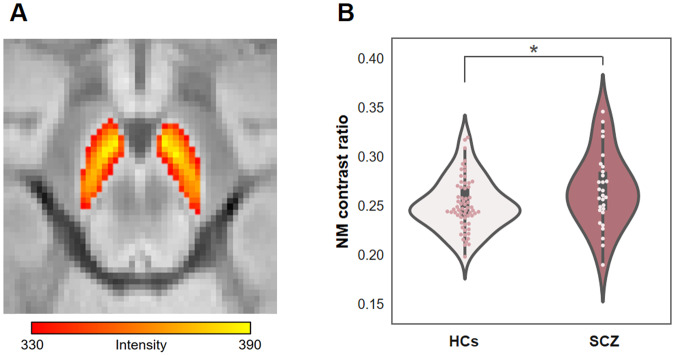
Neuromelanin (NM) in schizophrenia patients (SCZ). **A** A high-intensity area in the substantia nigra in the NM-MRI scan of a representative subject. **B** In SCZ, the NM contrast ratio was higher than that in healthy controls (HCs). (*) indicates significance at *p* < 0.05.

We examined group differences in NM CR between schizophrenia patients (mean = 0.27, 95% CI = 0.25–0.28) and healthy controls (mean = 0.25, 95% CI = 0.25–0.26) (Fig. 2). We calculated NM CR using the average intensity of the SN and CC regions. For the SN target region, we computed intensity values using the upper quartile voxel values to identify high-intensity NM-containing voxels. There was a statistically significant increase in NM CR in schizophrenia patients (*t* = −2.12, *p* = 0.037, Cohen’s *d* = 0.43).

### Aberrant FST subnetworks

**Fig. 3:**
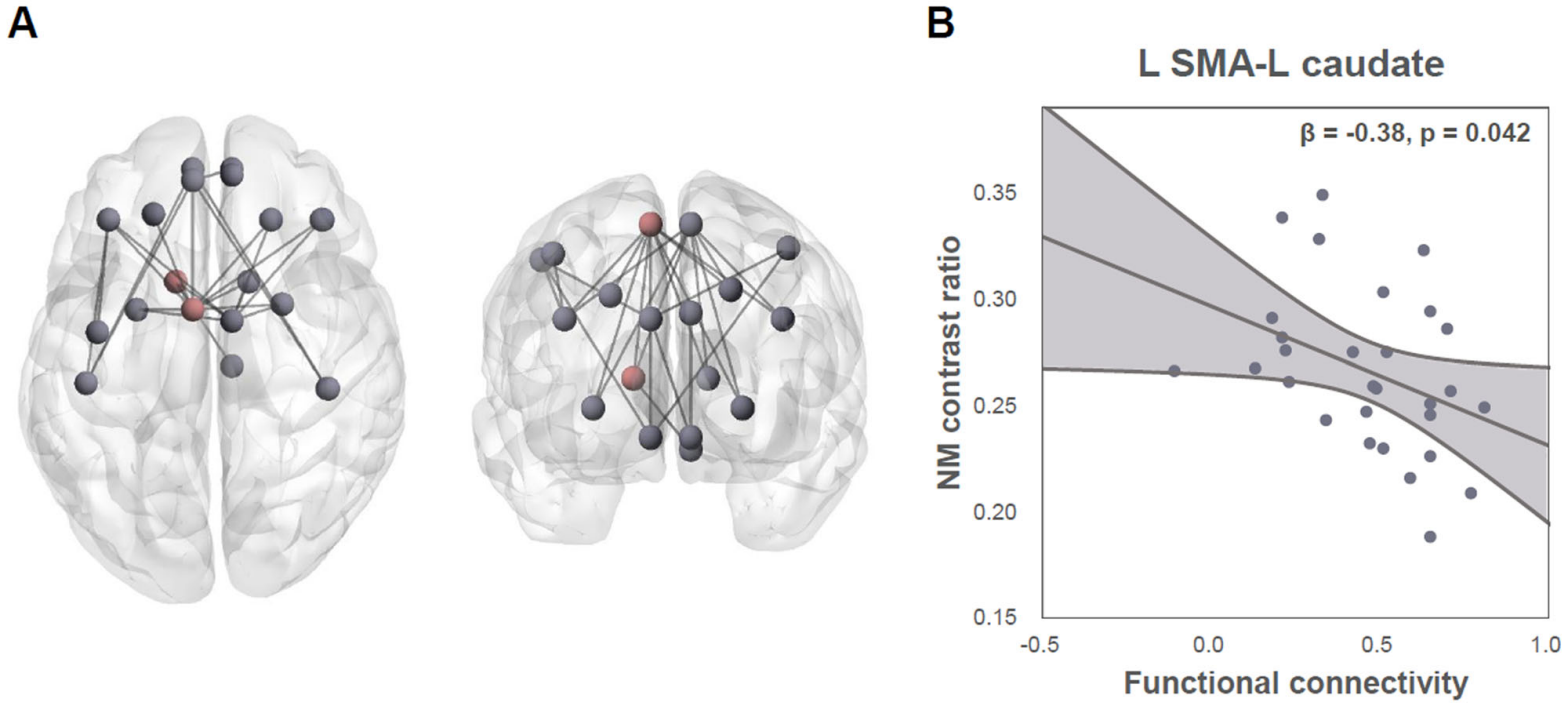
Subnetwork connectivity and neuromelanin (NM) in schizophrenia patients. **A** There was a hypoconnected fronto-striato-thalamic circuit subnetwork in patients, which consisted of 18 nodes. Among them, left supplementary motor area (SMA)-left caudate connectivity (dark red) showed a significant association with the NM contrast ratio. **B** Lower left SMA-left caudate functional connectivity was associated with a higher NM contrast ratio.

We found a hypoconnected FST subnetwork in patients compared to healthy controls (FWER-corrected *p* = 0.014), which consisted of 18 nodes and 26 edges (Table 2, Fig. 3). The hypoconnected subnetwork included cortico-cortical, striato-cortical, and nigro-striatal connections. In contrast, there was no significant hyperconnected subnetwork in schizophrenia patients. In the validation analysis, suprathreshold connections at the chosen primary threshold were replicated across different thresholds, which indicates the robustness of our result (Supplementary Fig. 1).

**Table 2:** Hypoconnected FST subnetwork in patients with schizophrenia.

Individual connections		Connectivity strength		*t*
		HCs	SCZ	
L PreCG	L MFG	0.69	0.56	3.19
L SFG	L SMA	0.67	0.54	2.78
R SFG	L SMA	0.63	0.49	2.65
	R SMA	0.65	0.50	3.18
L MFG	L SMA	0.66	0.50	3.64
	R SMA	0.57	0.42	3.11
	L PoCG	0.53	0.41	2.54
R MFG	L SMA	0.59	0.48	2.56
	R SMA	0.64	0.53	2.63
L SMA	L SFGmedial	0.52	0.38	2.56
	L Caudate	0.59	0.46	2.73
	R Caudate	0.55	0.43	2.60
	L Putamen	0.60	0.44	3.32
	R Putamen	0.57	0.40	3.28
R SMA	L Caudate	0.52	0.39	2.65
	R Caudate	0.52	0.39	2.61
	L Putamen	0.59	0.42	3.33
	R Putamen	0.59	0.42	3.39
L SFGmedial	L PFCventmed	0.79	0.66	4.07
	R PFCventmed	0.73	0.62	3.15
	L PoCG	0.39	0.21	3.01
	R PoCG	0.36	0.20	2.54
R SFGmedial	R PFCventmed	0.74	0.64	2.77
L PFCventmed	L PoCG	0.39	0.23	2.62
	R PoCG	0.35	0.19	2.64
L Caudate	R SNc	0.27	0.15	2.62

*HCs* healthy controls, *SCZ* schizophrenia patients, *PreCG* precentral gyrus, *SFG* superior frontal gyrus, dorsal lateral, *MFG* middle frontal gyrus, *SMA* supplementary motor area, *SFGmedial* superior frontal gyrus, medial, *PFCventmed* superior frontal gyrus, medial orbital, *PoCG* postcentral gyrus, *SNc* substantia nigra pars compacta.

### Association between NM and FST connectivity

We predicted the NM level from the subnetwork connectivity in schizophrenia patients using multiple linear regression analysis (Fig. 3). Among the 26 connections of the aberrant FST subnetwork, the left SMA-left caudate connection was selected as a significant predictor (*F* = 4.54, *p* = 0.042), while the other connections were excluded from the model. The model explained 11.20% of the variance (adjusted *R*^2^ = 0.11), and there was a negative effect between the predictor and dependent variable (β = −0.38). In contrast, there was no subnetwork connectivity that significantly predicted the NM level in healthy controls.

## Discussion

In this study, we confirmed changes in NM-MRI signal and functional connectivity of the FST circuit and found a significant association between these changes in schizophrenia patients. In the circuit, functional connectivity impairment was primarily in the cortex, striatum, and substantia nigra regions, among which left SMA-left caudate connectivity was associated with NM. This association indicates that the two pathological changes are interrelated in schizophrenia pathophysiology. Moreover, given that NM-MRI provides a proxy measure for dopamine activity, these results suggest that dopamine underlies the connectivity changes in the FST circuit.

We observed a higher NM-MRI signal in schizophrenia patients than in healthy controls; a higher signal indicates increased NM accumulation in the SN^22^. This result is consistent with the findings of previous NM-MRI and postmortem studies that reported increased NM levels in the SN in schizophrenia patients^44–47^. In dopamine metabolism, excessive dopamine induces an increase in NM synthesis^26^. Moreover, previous multimodal studies reported a positive correlation between NM-MRI signals and PET dopamine measures, such as dopamine release and dopamine receptor availability^22,28,29^. In this context, the higher NM-MRI signal in our study may suggest an increase in dopaminergic function. As mentioned above, increased dopamine is one of the most prominent abnormalities in schizophrenia^8^.

Our study found a hypoconnected FST circuit subnetwork in schizophrenia patients, which includes the cortex, striatum, and substantia nigra regions. Consistent with this, previous studies have shown hypoconnectivity in cortico-cortical, striato-cortical, and nigro-striatal connections in schizophrenia patients^12,48,49^. Preclinical models of schizophrenia pathophysiology explain this hypoconnectivity: spontaneous phasic dopamine release in schizophrenia patients leads to increased noise in dopamine signalling in the striatum, which in turn reduces functional connectivity in the FST circuit^8,50^. In contrast, some studies have reported increased connectivity, such as in the thalamic subnuclei, in schizophrenia patients^13,14^. These connections may not have been found in our study due to network-level statistics or the inclusion of the total thalamus rather than the subnuclei.

The dysconnected FST subnetwork included many striatal connections. The striatum is where dopaminergic projections from the midbrain arrive, and connectivity changes in this region are among the most consistent findings in schizophrenia^8^. Specifically, striatal connectivity alterations were prominent in the caudate^14^. In addition, cortical connectivity changes based on the aberrant striatum regions were primarily in the sensorimotor cortex^12^. These findings are in line with our results showing multiple caudate-sensorimotor cortex dysconnectivity. In our study, we also found connectivity changes in the nigro-striatal pathway. Previous studies suggested that reduced nigro-striatal connectivity may reflect reduced tonic activity and increased phasic activity in the pathway, given that rs-fMRI measures low-frequency fluctuations that are central to tonic activity^12^.

The FST circuit connectivity significantly predicted NM levels in schizophrenia patients. More specifically, lower left SMA-left caudate connectivity was associated with higher NM levels. Considering that NM levels reflect dopamine function, this result is consistent with previous findings showing an association between dopamine measures and striatal connectivity in schizophrenia patients^14,19,20^. In addition, our results of the association primarily identified in the SMA-caudate connection correspond with regional characteristics. According to recent neurochemistry studies, dopaminergic aberrations were greater in the dorsal striatum than in the limbic striatum^5,51,52^. The dorsal striatum encompasses the caudate region and receives cortical projections mainly from the SMA, motor area, and dorsolateral prefrontal cortex^10^. Taken together, this study adds to other evidence suggesting that midbrain dopamine changes underlie aberrant connectivity of the FST circuit. In contrast, healthy controls showed no correlation between NM and connectivity. NM is formed from excessive dopamine^26^, and its signal level and variability were relatively lower in healthy subjects. This low variability may have caused the absence of correlation.

This study suggests the possibility of using NM-MRI for various investigations of the dopamine system. For example, treatment response studies reported a linkage between responsiveness and dopamine and noted a need for using molecular imaging for response prediction^53–56^. PET is a typical molecular imaging method but has limitations in clinical settings due to its invasiveness, cost, etc. NM-MRI is safer in terms of radiation exposure and has better accessibility. In this study, we confirmed that NM-MRI results are consistent with existing molecular imaging results and examined the association between NM-MRI signals and functional connectivity for the first time. Overall, this study has implications for the future use of NM-MRI to examine dopamine function in research and clinical practice.

The main strength of this study is that it examined the association between FST circuit individual connectivity and dopamine system molecules at the network level in schizophrenia patients. Furthermore, this study showed the results of investigating the dopamine system using NM-MRI, a promising molecular imaging method. This study has several limitations. Schizophrenia patients in our study were medicated. Although there was no significant correlation between the analysis results and clinical variables, including medication (Supplementary Table 1), the effect of medication should be considered when interpreting the results. Antipsychotics affect the dopamine system, which may have altered NM levels and functional connectivity. Second, some studies reported different patterns of connectivity among subregions in the FST circuit, particularly in the thalamic subregions^14^. Future studies using higher resolution are needed to examine the subregions. Next, considering that psychosis may affect only a subregion of the SN^22^, the manual segmentation method may have diluted the effect size. Future studies would be advised to consider automated methods for greater anatomical precision. Finally, our association results do not indicate a causal relationship. Further investigations, for example, using sophisticated models, are required to determine causality within the pathophysiology.

We demonstrated a negative association between NM and FST circuit connectivity in schizophrenia patients using NM-MRI and fMRI. This shows a linkage between abnormal neurochemistry and functional abnormalities in the pathophysiology of schizophrenia. Furthermore, considering that the NM-MRI signal reflects dopamine function, our results suggest that dopamine underlies connectivity changes in the FST circuit. Together, these results expand our understanding of schizophrenia pathophysiology by providing experimental evidence supporting the dopamine hypothesis. In addition, this study has implications for the future use of NM-MRI in studies on the dopamine system.

### Supplementary information

Supplemental Material

## Data Availability

The data supporting the findings of this study are available from the corresponding author upon reasonable request.
