# Supplementary material for: Fronto-striato-thalamic circuit connectivity and neuromelanin in schizophrenia: an fMRI and neuromelanin-MRI study
Source: Schizophrenia (Heidelb). 2023 Nov 10;9(1):81. doi: 10.1038/s41537-023-00410-8 (PMC10636101; doi:10.1038/s41537-023-00410-8)
Supplement: Supplementary file 1 — Supplemental Material [file 41537_2023_410_MOESM1_ESM.docx]

**Fronto-striato-thalamic circuit connectivity and neuromelanin in schizophrenia: an fMRI and neuromelanin-MRI study**

**Supplementary Material**

Sunah Choi, Minah Kim, Taekwan Kim, Eun-Jung Choi, Jungha Lee, Sun-Young Moon, Sang Soo Cho, Jongho Lee, Jun Soo Kwon


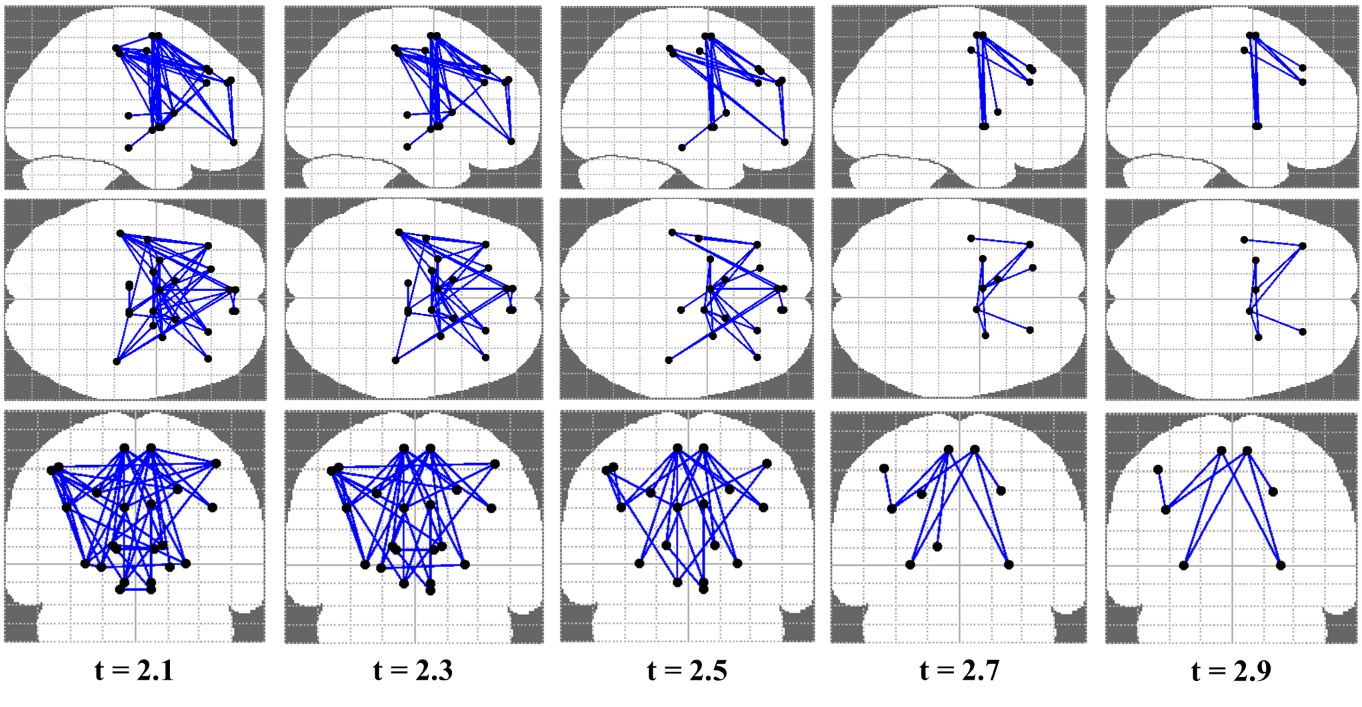


**Supplementary Figure 1.** Subnetwork connectivity across different primary thresholds (t = 2.1-2.9 corresponding to p = 0.04-0.005).

**Supplementary Table 1.** Correlation with olanzapine equivalent dose

|  |  | **Olanzapine equivalent dose** | |
| --- | --- | --- | --- |
|  |  | **Spearman's rho** | **p** |
| **Subnetwork functional connectivity** | |  |  |
| L PreCG | L MFG | -0.08 | 0.663 |
| L SFG | L SMA | -0.13 | 0.495 |
| R SFG | L SMA | -0.06 | 0.747 |
|  | R SMA | 0.02 | 0.917 |
| L MFG | L SMA | -0.20 | 0.296 |
|  | R SMA | -0.11 | 0.580 |
|  | L PoCG | -0.04 | 0.841 |
| R MFG | L SMA | -0.27 | 0.161 |
|  | R SMA | -0.11 | 0.566 |
| L SMA | L SFGmedial | -0.24 | 0.204 |
|  | L Caudate | -0.04 | 0.822 |
|  | R Caudate | -0.10 | 0.620 |
|  | L Putamen | -0.07 | 0.720 |
|  | R Putamen | -0.22 | 0.256 |
| R SMA | L Caudate | 0.03 | 0.884 |
|  | R Caudate | -0.09 | 0.637 |
|  | L Putamen | -0.02 | 0.928 |
|  | R Putamen | -0.08 | 0.690 |
| L SFGmedial | L PFCventmed | 0.12 | 0.519 |
|  | R PFCventmed | 0.13 | 0.497 |
|  | L PoCG | -0.15 | 0.453 |
|  | R PoCG | -0.04 | 0.841 |
| R SFGmedial | R PFCventmed | 0.09 | 0.646 |
| L PFCventmed | L PoCG | -0.17 | 0.381 |
|  | R PoCG | -0.09 | 0.652 |
| L Caudate | R SNc | 0.30 | 0.118 |
| **Neuromelanin contrast ratio** | | 0.20 | 0.293 |

PreCG: precentral gyrus; SFG: superior frontal gyrus, dorsal lateral; MFG: middle frontal gyrus; SMA: supplementary motor area; SFGmedial: superior frontal gyrus, medial; PFCventmed: superior frontal gyrus, medial orbital; PoCG: postcentral gyrus; SNc: substantia nigra pars compacta.
